# A critical account of the policy context shaping perinatal survival in Nepal: policy tension of socio-cultural versus a medical approach

**DOI:** 10.1186/s12913-019-3979-3

**Published:** 2019-03-14

**Authors:** Mohan Paudel, Sara Javanparast, Gouranga Dasvarma, Lareen Newman

**Affiliations:** 1 Initiative for Research, Education and Community Health-Nepal, Kathmandu, Nepal; 2 Southgate Institute of Health, Society & Equity, Flinders University, Adelaide, Australia; 3 College of Humanities, Arts and Social Sciences, Flinders University, Adelaide, Australia; 4 Education Arts and Social Sciences Divisional Office, University of South Australia , Adelaide, Australia

**Keywords:** Perinatal survival, Policy, Strategy, Newborn, Inequity, Nepal

## Abstract

**Background:**

Nepal formulated a range of policies related to maternal and neonatal survival, especially after the year 2000. Nevertheless, Nepal’s perinatal mortality remains high, particularly in disadvantaged regions. Policy analysis can uncover the underlying values, strategies and policy formulation processes that shape the potential to reduce in-country health inequities. This paper provides a critical account of the main policy documents relevant to perinatal survival in Nepal.

**Methods:**

Six key policy documents covering the period 2000–2015 were reviewed using an adapted framework and were analyzed through qualitative content analysis.

**Results:**

The analysis shows that the policies focused mainly on the *system*: improvement in provision of birthing facilities; targeting *staff* (Skilled Birth Attendants) and *health service users* by providing cash incentives to staff for bringing patients to services, and to users (pregnant women) to attend health institutions. Despite a growing focus on saving women and newborn babies, there is a poor policy focus and direction on preventing stillbirth. The policy documents were found to emphasize tensions between birthing at home and at health institutions on the one hand, and between strategies to provide culturally appropriate, woman-centered care in communities and medically orientated services on the other. Policies acknowledge the need to provide and address woman-centered care, equity, social inclusion, and a rights-based approach, and identify the community based approach as the mode of service delivery. Over and above this, all policy documents are aimed at the national level, and there is no specific policy direction for the separate ecological, cultural or geographic regions such as the mountainous region, which continues to exhibit higher mortality rates and has different cultural and demographic characteristics to the rest of Nepal.

**Conclusions:**

To better address the continuing high perinatal mortality rates, particularly in disadvantaged areas, national health policies should pay more attention to the inequity in healthcare access and in perinatal outcomes by integrating both stillbirth prevention and neonatal survival as policy agenda items. To ensure effective translation of policy into practice, it is imperative to tailor the strategies according to acknowledged policy values such as rights, inclusion and socio-cultural identity.

## Background

Despite the decades old global focus on improving maternal, infant and under-5 health outcomes in developing countries, perinatal mortality (which includes stillbirth and neonatal mortality in its extended definition) has remained largely overlooked [1]. Perinatal mortality has high social and economic costs, and is a marker of existing inequalities in countries and their communities. About 2.7 million neonatal deaths and 2.6 million stillbirths are estimated to occur annually across the world [2, 3]. Of the total estimated neonatal deaths, 2 million occur in the first week of life. Nearly 99% of both stillbirths and neonatal deaths occur in developing countries, including three-quarters in South Asian and African countries. Failure to further improve birth outcomes is estimated to cause 116 million deaths, 99 million with disability or loss of potential, and additional millions of adults with increased risk of later-life non-communicable diseases from being born with Low Birth Weight [4, 5].

Nepal has made good progress in reducing maternal and under-five mortality rates over the years [6, 7] with much remaining still to be done on the reduction of perinatal deaths—both stillbirths and neonatal deaths. The neonatal death rates as identified by Nepal’s 2011 Demographic and Health Survey are reported to be among the highest in the world (neonatal mortality: 33 per thousand livebirths, and perinatal mortality: 37 per thousand births), [8] with one of the highest neonatal mortality differentials according to income inequality and geographical location [9]. It is estimated that Nepal could potentially have a reduction of 46% in its neonatal mortality rate if the existing income inequalities were removed [4]. Nepal’s National Demographic and Health Surveys of 2006 and 2011 identified a stable national neonatal mortality rate at 33 per thousand livebirths, compared with the global average of 21 per thousand livebirths [8, 10]. Although the most recent Demographic and Health Survey conducted in Nepal in 2016 reported a reduction in neonatal mortality, it still identified high perinatal mortality (36/1000 pregnancies) in rural areas including the mountains, and persistent geographic differentials in neonatal death rates, with the mountains reporting the highest rates [11, 12]. Currently, the neonatal mortality rate in Nepal’s mountain region is 35 per thousand live births [11], which is more than the neonatal mortality rates of the Sub-Saharan Africa (28/1000) livebirths [13]. Very similar patterns are also reported in perinatal mortality, both nationally and in the mountainous region, despite ambiguities in definition and omissions in reporting of stillbirths in these regions.

Analysis of health policies is crucial for understanding their influence on health systems, and their focus and impact on population health [14, 15]. Hafner and Shiffman [16], while discussing the influence of global health policy changes in strengthening health systems, argue that population health and equity are affected by the limitations of a vertical medical approach (disease oriented initiatives), adverse effects of global health initiatives on local health systems, and bottlenecks in weak health systems. In this context, other researchers [17–19] recommend that an increasing focus is required to examine the policy strategies, the policy process and the use of evidence in policy formulation. Therefore, the focus of the current paper on policy review is crucial to examine the underpinning values and strategies and in order to derive insights into potential to improving healthcare delivery, in this case, to understand the potential to reduce in-country inequities in improving poor perinatal survival in Nepal.

Up until the year 2000, Nepal did not have specific policy strategies focusing on maternal and newborn health. After 2000, Nepal experienced several key changes in policies related to perinatal survival. Examining the policy documents is therefore one way to provide insights into why poor perinatal survival rates continue both at national, ecological and sub-national levels.

This paper commences with an overview of the historical development of policy relevant to maternal and perinatal health in general, before explaining how the focus in Nepal after 2000 began to move more towards improving newborn survival. Each document is then separately reviewed, providing details of the document, strategies and intended outcomes, and values and principles ingrained in the document.

## Method

This paper is extracted from the first phase of a much larger, qualitative research project entitled ‘Socio-cultural and healthcare context of perinatal survival in rural mountain villages of Nepal’ [20]. As its first phase, the larger research project comprised a review of policy related documents in, and a qualitative research fieldwork conducted in a mountain district of Nepal in its second phase. Research ethics approval for the project was obtained from the Social and Behavioral Research Ethics Committee of Flinders University in South Australia and from the Nepal Health Research Council.

### Document identification and selection

**Fig. 1:**
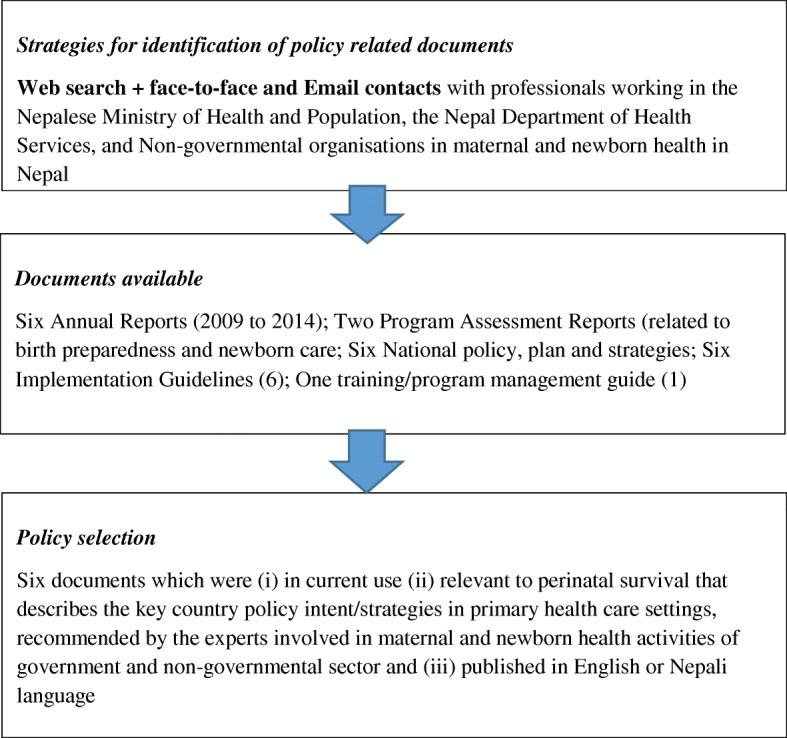
Document review flow diagram. Source: Author created based on document search

Previous employment in Nepal for nearly 6 years in the public health sector enabled the first author (a Nepali national) to access professional networks to know and identify the key policy related documents currently effective in Nepal. Figure 1 describes the process of document identification and selection. This stage helped to further reflect on the objective of this paper and consult experts working in newborn and maternal health in government and non-governmental sectors in Nepal. Drawing on this knowledge base, the decision was made to individually review six key documents recommended as the most appropriate and most effective in shaping Nepal’s policy level response to improve perinatal survival at primary healthcare level. These 6 were selected from a pool of 21 documents which focused broadly on health service delivery, where the 6 focused particularly on maternal and newborn health and were also the more recent documents in use.

### Review process

This review utilized a qualitative content analysis (directed approach) as suggested by Hsieh and Shannon (2005), guided by an adapted framework **(**Table 1**)** as an initial framework to code contents in the Nepalese documents. The framework and policy review process were developed by drawing on insights from Walt and Gilson (1994) and Newman et al. (2006a, 2006b). The first and second rows in Table 1 provide context and process, and the third, fourth and fifth rows provide values and content identified by the policies in Nepal. While reviewing each document, the five categories of the framework became nodes in the NVivo (Version 10.00) software, and the bullet points under each category became the areas of interests and constant questions to code into each particular category. To describe the context of how policy making evolved in Nepal, background papers related to the policy documents and the previous versions of the documents were also utilized.

**Table 1:** Document Review Framework

Key areas	Review questions
*1. The document*	• Purpose (what is the main purpose of the document?) • Type of document (is it a policy/strategy/planning document? Progress/assessment report? Training/guideline? • Focus on newborn or stillbirth or both (does it mention newborn, perinatal care explicitly?) • Geographic focus (what type of geographic area is the key focus of the document--National? Topography specific? Development region?)
*2. Document development*	• Process (what led to the formulation of this document? Why was the need felt to formulate this document?) • Developers (which department, section formulated it?) • Stakeholders (who were the stakeholders involved?)
*3. Values, definitions and language*	• Social or Medical focus (what is the key focus of the policy?) • Key perspective/approach in deciding to reach perinatal care (right based, woman-centered, gender, etc.) • Equity groups (have they targeted vulnerable groups or disadvantaged groups e.g. teenage mothers, scheduled castes/indigenous and ethnic minorities?)
*4. Health outcomes and health access*	• Prevention of stillbirths • Prevention of neonatal deaths • Access to care during pre-pregnancy, pregnancy, delivery, postpartum period
*5. Strategies for action*	• The strategies in providing perinatal and neonatal care such as at home, in community, and in health facility • Care across the continuum from pre-pregnancy to postpartum • Integration of perinatal and neonatal care with maternity and child survival and other interventions • Inter-sectoral collaboration, collaboration across departments • Other government departments and agencies involved such as UN, bi-lateral agencies, INGOs • Specific target groups

Source: Adapted from Newman [53], Newman [54] Walt and Gilson [15]

## Results

### Perinatal survival—Low focus before 2000

The document review showed that prior to 2000, perinatal survival was not a focused strategic outcome anywhere in policy documents in Nepal’s healthcare system. Nepal’s response to address the health of women and children in general dates back to 1989 with the introduction of a voluntary network of Female Community Health Volunteers as a foundation of community health [21]. Currently, there are over 50,000 female volunteers which the national health policy [22] considered a major pillar in improving healthcare for women and children, but their intention was mainly to address ongoing high maternal and under-five mortality. However, the first ever programmatic response to high maternal and child mortality rates can be traced back to the Family Planning and Maternal and Child Health initiative of 1968 [23]. Later, in 1998, Nepal formulated the National Reproductive Health Strategy which included child health and safe motherhood as its components, and endorsed a basic standard of reproductive care from different levels of health facilities [24]. Yet, nowhere did this strategy specifically talk about perinatal survival as such. Its concern was only around whether a mother could be saved, because a large number of women were dying during pregnancy, childbirth and the postnatal period. Before 2000, the maternal and under-five death tolls in Nepal were among the highest in the world [8, 25, 26]. Safe motherhood became the government’s priority program, and Nepal developed its first Safe Motherhood Policy in 1993 [27] with the main and urgent focus to save women’s lives during childbirth and within the postnatal period. Early infant deaths did not come into policy documents when Nepal was grappling with one of the highest maternal and under-five mortality rates in the world.

### Policies after 2000— ‘Newborn focused’ but low priority in addressing stillbirth

Survival of newborns did not become a major strategic priority until Nepal developed the 2004 National Neonatal Health Strategy [28]. Still, in all policies, prevention of stillbirths received less priority than in preventing neonatal deaths after birth. The driving force behind Nepal’s policy response to prevent neonatal deaths is the international initiative- Millennium Development Goal Four - when Nepal realized that reaching under-five and infant mortality targets was not possible without first addressing its high prevalence of neonatal mortality. Of the six key documents considered effective in impacting perinatal survival in Nepal, the National Neonatal Health Strategy 2004 is considered the most specific in addressing newborn healthcare [28]. The other policies are:

- National Policy on Skilled Birth Attendants, 2006 (Supplementary to Safe Motherhood Policy, 1998, [29];
- National Safe Motherhood and Newborn Health Long Term Plan (2006–2017) [27];
- Mother’s Protection Program-Implementation Guideline, 2013 (revision on Safe Delivery Incentive Guideline, 2007 and 2009, [30];
- Maternal and Perinatal Death Surveillance and Response (MPDSR) Guideline, 2014 [31] and;
- Community Based Integrated Management of Neonatal and Childhood Illness (Program Management Module, 2015) [32].

**Fig. 2:**
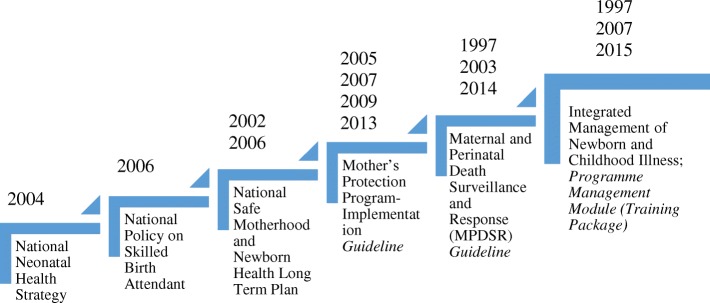
The selected documents and the update versions according to timeline. Source: Author created based on the document review

It can be seen that Nepal has been focusing on updating these policy documents since 2000. Figure 2 illustrates the policies and their subsequent revisions.

Guided by the adapted framework (as per Table 1), the paper now proceeds to provide a detailed analysis of individual documents. The analysis consists of descriptions of the content and focus of each of these six documents, their development process, their key values and motivations, and their health outcomes and strategies considered to influence perinatal survival in the country.

#### National Neonatal Health Strategy, 2004

Nepal’s National Neonatal Health Strategy [28] is considered as the first strategic response focusing specifically on the newborn at the country level. This strategy was formulated with the aim to prevent neonatal deaths when more than 30,000 newborns were dying every year in Nepal (p. 2)^1^ which translates into approximately three to four neonatal deaths an hour, half of which were in the mountains. This document was formulated when the situation was marked by a high proportion of home births, poor postnatal care, a poorly functioning referral system, and unattended obstetric and neonatal complications (pp. 2–3).

Regarding the underpinning values of this policy, it is stated in the front of the policy document that the then secretary of the Nepalese Ministry of Health and Population acknowledges *access to care* and *survival as the greatest right* of every vulnerable newborn:

Every vulnerable Nepali newborn has the greatest right to be taken care of and therefore we have to immediately invest resources to improve their health and survival (p. ii) (Secretary, Ministry of Health and Population [Nepal])

The policy recognized the strength of addressing problems in *mothers and babies’ health in a continuum:*

The mother and her baby should be treated as one entity and to be successful; any range of interventions that seek to prevent perinatal and neonatal deaths must address both maternal and neonatal factors. (p.1)

Although it mentioned that priority should be given to remote and disadvantaged areas, it did not explicitly identify regional variation in neonatal death rates and did not provide any regional/ecological specific priorities. Hence, the significantly higher neonatal death rates in the mountain areas are not specifically highlighted, nor are mountain-specific strategies outlined to address them, except generally stating that priority should be given to remote and rural areas.

The policy considered a set of evidence based and proven interventions such as immediate newborn care with drying, delayed bathing, skin to skin contact, breastfeeding and treatment of infections. This policy sets its immediate focus on addressing complications of mothers and babies for immediate impact:

… proven interventions addressing causes of maternal and neonatal complications at family and community levels will be the primary focus for immediate impact. These interventions will require the establishment of a chain of care linking families and communities with the health system. (p. 2)

The main policy intention remained focused on promoting institutional births and prevention of neonatal deaths during childbirth or shortly after birth. This policy also devised strategies to institutionalize provision of newborn care from Nepal’s healthcare system. To do this, the policy set neonatal care at four different levels: (1) home/community level, (2) primary healthcare level, (3) district hospital level and zonal, (4) regional and central hospital level (p. 12). It sets forth five key intervention areas related to birth registration; behaviour change engaging mothers, husbands and mothers-in-law; healthcare delivery by Skilled Birth Attendants (SBAs); ensuring supplies and logistics; and research in areas of quality of care including verbal autopsy studies (pp. 4–7).

This national strategy also discussed the establishment of a well-functioning referral mechanism for sick newborns, care for non-sick low birth weight babies, and management of newborn infections with appropriate antibiotics from village health institutions. At the hospital level, it included additional activities such as management of obstetric and newborn complications, intravenous drug administration, perinatal death audits and training and supervisory activities of staff working at peripheral health institutions.

#### National Policy on skilled birth attendant, 2006

Nepal introduced a Skilled Birth Attendant (SBA) policy in 2006 [29] to specifically address high maternal and neonatal mortality. This policy is supplementary to the Nepal Safe Motherhood Policy, 1998. The Ministry of Health and Population formed a policy advisory group in May 2006 which then developed the SBA policy in 2006. Nepal considered formulation of this policy imperative in the context that only 13% of birthing women were attended by health workers. Compared with SBAs, Maternal and Child Health Workers (MCHW) and Auxiliary Nurse Midwives (ANMs) were identified as being ineffective in reducing the number of maternal and neonatal deaths (p. 2). One of the main gaps noted in relation to MCHWs and ANMs concerned lack of professional competency, particularly to manage obstetric complications. This policy therefore set out to address the lack of access to life-saving emergency obstetric care to women in villages across the country.

The policy valued woman-friendly care during pregnancy and birthing, defined as culturally sensitive and affordable care. Although it did not specifically mention the mountain areas, it highlighted the priority to be given to the poor and under-served areas.

… it is important to encourage women to deliver in facilities with skilled attendants with access to Emergency Obstetric Care (EmOC). This will require 24 hours a day and 7 days a week, women-friendly services that are culturally sensitive and affordable to all families, especially those in poor and underserved areas. (p.1)

The policy stood on the research evidence that improving access to skilled attendants at birth prevents a large proportion of maternal and neonatal deaths. Citing the Nepali context where the majority of women were still giving birth at home, the policy explicitly conflated skilled attendance with institutional births and treatment of obstetric complications.

The strategies focused mainly on recruitment of SBAs and their deployment. The policy highlighted the need for creating an enabling environment for SBAs working in the periphery by ensuring regular supportive clinical supervision and medical supplies, effective partnership in the work environment with other health workers, and living accommodation and support from the local facility management committee.

The SBA will work in close partnership with other essential health care providers at community level and be supported and guided by a strong District Health Team that has the capacity to deal effectively with emergency obstetric complications … . Secretary of Ministry of Health and Population (p. i)

The policy also outlined the expansion of birthing units in health institutions, and encouraged NGOs and communities to establish community based birthing units at the local level (pp. 8–9).

#### National Safe Motherhood and newborn health long term plan (2006–2017)

The purpose of the National Safe Motherhood & Newborn Health Long Term Plan (2006–2017) [27] was to increase healthy practices and to improve the quality of professional care for both mothers and their newborn babies. The document was prepared in consultation with both government and non-governmental stakeholders at the central level. The formulation of the document evolved through three stages: (i) first preparatory meeting in November 2005; (ii) first workshop in January 2006 after consultants’ review of existing policies, gaps and development of a background paper for revision; and (iii) second workshop in March 2006 which set the detail of outputs and activities across various thematic groups.

This plan is the second version of Nepal’s National Safe Motherhood Long Term Plan (2002–2017) [33]. This version incorporated a response to some of the urgent changes at the time such as the MDGs and the Nepal government’s focus on neonatal health, increasing the numbers of SBAs at births, and the Health Sector Strategy: An Agenda for Reform [34]. This reform strategy was to ensure a system that provides access to essential health services to poor and vulnerable population; to develop capacity of local bodies for participatory and accountable management of health institutions, and to build partnership and mobilize NGOs and the private sector in maternity service delivery. This second version was produced to also incorporate the first ever MDG country progress report 2005 [35] which came after the first long-term plan introduced in 2002 [36]. Compared with the first version, this plan strongly prioritized SBA mobilization to reduce both maternal and neonatal mortality.

The National Safe Motherhood and Newborn Health Long Term Plan (2006–2017; NSMNH-LTP) was the first national plan to introduce a strongly stated focus on equity and social inclusion:

Equity issues in access and utilization of safe motherhood and neonatal health (SMNH) services are not mentioned in the original NSMLTP and are of critical importance if the most needy members of society are to be targeted and the MDGs achieved. (p. 2)

Another difference of this document from previous documents is that this document acknowledged the role of multiple sectors to ensure equity and access to care in maternal and newborn health:

Since safe motherhood and newborn health are not purely health issues, they warrant a multi-sectoral approach and the role of other sectors is particularly important in enhancing access and promoting equity. (p.1)

The plan also duly recognized the needs of women to be understood in complex social contexts:

The needs of women are treated as paramount throughout the NSMNH-LTP, not simply as individuals, but as members of families and communities*.*

The plan set eight strategic outputs to ensure progress in the health of mother and babies. These were: equity and access; delivery of quality maternal and newborn care; public private partnership; decentralization; and human resource development, mainly focusing on training of SBAs; information management; physical asset management and procurement; and finance such as financial safety nets. The plan also emphasized the need to understand local knowledge about the context of maternity and newborn care:

Activities will advantageously use local knowledge, perceptions and values, relevant traditional practices, preferences and beliefs to enhance knowledge and awareness and will be sensitive to conflict issues. (p. 7)

The plan identified access in a broad sense that would consider not only physical and financial access but also the cultural and behavioural aspects of service providers:

Access embraces financial, institutional and infra-structural factors including, but not limited to, funding, transportation and education. It also relies upon positive and welcoming service provider attitudes, trust, honesty, responsiveness, accountability and quality service delivery both at established facilities and through outreach programmes. (pp. 7-8)

The service delivery output of the plan emphasized reaching socially excluded groups, and encouraged the 24-h availability of skilled staff and district-specific strategies to increase service access:

At service level, efforts to improve the effectiveness of the system will focus on ensuring 24-hour availability of skilled staff with essential drugs and equipment, good community and inter-facility linkages and feedback systems to promote further improvements. Remote areas present an even greater challenge and require additional focused efforts, which will be covered by district specific strategies. (p.10)*.*

With regards to public-private partnerships, the plan sought increasing involvement of NGOs, private sector hospitals and academic institutions. In decentralization, the plan sought to ensure planning and supervising capacity with local government, ie the District Health Office and health institutions. Likewise, as a financial strategy, it sought to implement equity through creating financial safety nets for the poor and socially excluded. Regarding the information system, the plan highlighted collection and use of data according to ethnicity, caste and wealth.

#### Mother’s protection program, implementation guideline, 2013

The Mother’s Protection Program, Implementation Guideline, 2013 [30] is a successive revision of the previous guidelines of the Safe Delivery Incentive Programme (SDIP). The SDIP was first introduced in 2005, revised in 2007 and subsequently evolved as the Mother’s Protection Program, Implementation Guideline, 2009. This latest 2013 guideline was formulated by a working committee involving both district and central level experts within the Department of Health Services. The amendment to the previous version of 2009 was felt necessary to set criteria for types of health institutions to be provided with financial incentives, and to highlight that women should be given the first priority to have financial incentives before institutions and providers. The institutional incentive was to encourage health institutions to cover basic expenses including drugs and logistics, while the aim of monetary incentives to providers was to encourage them to ensure their 24-h availability for birthing services.

Advancing further on the Safe Delivery Incentive Programme (SDIP), the Mothers Protection Program ensured free maternity care from government health institutions and hospitals, and community and teaching hospitals throughout the country, not just limiting it to the 25 low Human Development Index (HDI) mountain districts. The SDIP 2005 and 2007 provided incentives for women to encourage them to attend institutional births in the 25 low HDI districts. This 2013 guideline ensured free maternity care throughout the country: (i) to reach the MDG maternal and child health targets; and (ii) to ensure the right to health as a fundamental constitutional right of every citizen in accordance with the provision of Nepal’s interim constitution 2006 (p. 4). The document also talked about the partnership approach in providing maternity and newborn care with private, teaching and community hospitals (p. 4).

The Safe Delivery Incentive Programme guideline 2005 and the first amended version of it (SDIP 2007) provisioned financial safety nets for improving access to maternity care. The amended 2007 version provisioned free maternity care in the all 25 low HDI remote mountainous districts. This also set a slightly higher travel incentive, about USD 15 (NRs 1500) to cover travel expenses from home to the institutional birth for women from the mountainous districts [37]. It set about USD 5 (NRs 500) for women in the plains areas, and about USD 10 (NRs 1000) to women in the hills districts. This is reasonable considering that women in the mountainous areas have considerably further to walk/travel to attend formal healthcare institutions. As also stated above, this Mother’s Protection Program Implementation Guideline continued the monetary travel incentive for giving birth in health institutions, and also expanded this to encourage pregnant women to attend health visits in the health institutions. However, this made the intention of the policy explicit on promoting institutional birth. It provisioned that women would receive the additional incentive for antenatal check-ups only when they continued attending for all the recommended four antenatal visits and institutional birth.

Mothers who came for four focused antenatal visits and also gave birth in health institution, will be provided rupees 400 [about 4 USD] from pregnancy and delivery incentive during discharge from health institution. (p. 6)

This guideline expanded the concept of birthing units (pp. 23–25) by setting specific criteria. These criteria included physical infrastructure and space with one separate birthing room; equipment; living quarters (accommodation) for the SBA; 24-h presence of a SBA including support staff; good referral network; friendly behaviour towards the woman and her visitors and the respect of a woman’s privacy while giving birth. In addition, the guideline made it necessary to report monthly on the number of obstetric complications managed (p. 20). The new obstetric reporting form included reporting to the district and central department of neonatal deaths, stillbirths and babies resuscitated for asphyxia management by each health institution. To encourage registration of birth and deaths, the guideline made a provision for a provider incentive for home births only upon submission of the report of the either birth or the death registration of a baby.

#### Maternal and perinatal death Surveillance and response (MPDSR), guideline 2014

The Maternal and Perinatal Death Surveillance and Response (MPDSR) [31] evolved from the Maternal and Perinatal Death Review (MPDR) which was initiated after first implementing Maternal Death Review (MDR) in selected hospitals in Nepal. The Maternal Death Review dates back to 1990 when Nepal’s Family Health Division (FHD) first implemented it in a national hospital in Kathmandu; this was implemented in technical support of the WHO. However, the Perinatal Death Review (PDR) component was introduced only in 2003, and then implemented for the first time in six Nepalese hospitals. By the year 2012, the MPDR had expanded to 21 hospitals across the country. In 2013, after revision of the PDR tool, this expanded to 42 hospitals that cover about half of the total hospitals throughout the country. After revision of the PDR tool, it evolved into the MPDSR form. Internationally, Nepal’s MPDSR is in accordance with the UN Global Strategy for Women’s and Children’s Health and the Commission on Information and Accountability (CoIA) (p. 4). This document is proof of the search by Nepal’s Ministry of Health and Population for a locally appropriate and viable mechanism to continuously strive to reduce maternal and perinatal deaths. The two key objectives of the document (pp. 4–5) were: (i) “To provide information that effectively guides immediate as well as long-term actions to reduce maternal mortality at health facilities and community and perinatal mortality at health facilities”; and (ii) “To count every maternal and perinatal death, permitting an assessment of the true magnitude of maternal and perinatal mortality and the impact of actions to reduce it”.

This guideline acknowledged the value of the life of every mother and every baby:

“MPDSR underlines the critical need to respond to every maternal and perinatal death, so that the information obtained from that death might be acted upon to prevent future deaths” (p. 3) … every death can provide information that can result in actions to prevent future maternal and perinatal deaths (p. 25)*.*

Strategically, this document aimed at linking the information system with the quality improvement process at a health institution level. The purpose was to enable real-time monitoring of deaths and assessment of the interventions employed.:

The notification of every maternal and perinatal death also permits the measurement of maternal mortality ratios and perinatal mortality and the real-time monitoring of trends that provide countries with evidence about the effectiveness of interventions. (p. 3)

The above statement identified notification of every death, but so far it prioritized notification and review of every maternal death occurring both at institutions and in communities; whereas, for perinatal deaths this applied only at institution level. Hence, it is likely to miss a considerable number of deaths occurring in communities, and more so in the remote mountainous areas which still have high perinatal deaths.

This guideline recommended that the MPDSR cycle comprise five key elements: case identification, information collection, analysis, recommendation for action, and evaluation. The case identification involved notifying any maternal deaths in the institution and community, and perinatal deaths in an institution. Likewise, the guideline proposed a committee for the review of maternal and perinatal death at each health institution level. The committee did not explicitly include any parent/family representative/s. The review tool comprised structured questions focusing predominantly on clinical details of the deceased baby, possible causes of deaths, and the healthcare procedures followed for treatment. It does not include parental views on causes for delayed care-seeking, nor does it focus on identifying socio-cultural aspects of perinatal deaths.

#### Community based integrated Management of Neonatal and Childhood Illness (program management module), 2015

Community Based Integrated Management of Neonatal and Childhood Illness (Program Management Module, 2015) [32] describes the most recently revised package of key interventions to address newborn survival in Nepal. This program package is considered the most recent update of Nepal’s continuous efforts to increase newborn survival, including children under-5 years. It focuses on the provision of improved care by health service providers including trained health volunteers in local communities.

This packaged ‘CB-IMNCI’ programme is the result of lessons learned over the last three decades from a range of previous programmatic interventions related to vertically implemented programmes on diarrhoeal disease control, acute respiratory infection (ARI), and integrated management of childhood illness (IMCI) which aimed to manage five major killer diseases of under-five children: malaria, malnutrition, measles, pneumonia and diarrhoea. The IMNCI was brought to community level as Community Based Integrated Management of Childhood Illness (CBIMCI) where female health volunteers became a key cadre for treating and referring children under-five, including newborns. The Community Based Newborn Care Program (CBNCP) from 2007 gave special focus to newborn care at home, community and peripheral health institutions.

The CBNCP comprised a package of key interventions to be delivered from health institutions and female community health volunteers (FCHVs). It included promotion of institutional births, social mobilization for health-related behaviour change mainly through FCHVs and mothers group; postnatal check-up visits for mothers and newborns; management of possible bacterial infections such as diarrhoea and pneumonia; management of low birth weight babies mainly by keeping them warm (e.g. via kangaroo care); prevention of hypothermia; and management of asphyxiated babies. This ‘CBNCP’ package was further revised, and is now its most recent form as ‘CB-IMNCI’.

The CB-IMNCI still emphasizes the newborn component, including the interventions of CBNCP, and additionally integrating the IMCI for effective management of problems for all under-5 s in one single package. The document’s key objectives (p. 12) are: (i) to reduce newborn morbidity and mortality by the promotion of immediate care of newborn babies; (ii) to reduce newborn morbidity and mortality by managing health problems of newborn babies; and (iii) to reduce under-five morbidity and mortality by managing their health problems.

Generally, the document discusses improving the quality of newborn care; extending care to communities; reaching marginalized and disadvantaged women/babies; strengthening the supply system; continuing research and investigations for programme improvement and positive behaviour change at home and community; and community participation in newborn care (p.12). However, the document does not specifically discuss how it will reach marginalized populations. In addition, despite being a recent document, it does not have any focus on stillbirths, though this is an equally serious concern [38].

Despite the intention of being community based as per its title, this document still focuses mainly on promotion of institutional births and strengthening of quality of care from health institutions to prevent neonatal deaths. The focus has been on strengthening the capacity of institutions to manage and treat newborn babies’ complications. The package has also envisioned expanding birthing centres to ensure quality childbirth and referral care for newborns with complications. Institutionalization has been a key focus, and for the near future it also envisions a new programme entitled Facility Based Integrated Management of Neonatal and Childhood Illness (p. 11). It has added a component which describes treatment of baby’s cord infections by using an antiseptic ointment, chlorhexidine. The package does not consider management of asphyxia as the local health volunteers’ job responsibility. However, asphyxia management was considered a major skill in CBNCP package.

Overall, a brief summary of key policy values and strategies of each of the documents described above is presented in Table 2.

**Table 2:** Summary of key values and strategies within the six main documents

Key values (approach, underpinning principles)	Strategies (strategic interventions)
National Neonatal Health Strategy 2004	
• *Access to care and survival as the greatest right* of every vulnerable newborn • Mothers and babies’ *health in a continuum from pre-pregnancy to postnatal* • A *linkage of care across home, community and health institution* • *Gender equality in newborn care*	• Focusing on proven interventions addressing causes of maternal and neonatal complications • Promoting institutional births and preventing newborn deaths during the process of childbirth or shortly after birth • Institutionalising provision of newborn care from Nepal’s healthcare system: (i) home/community; (ii) primary healthcare; (iii) district hospital; (iv) above the district hospital at zonal, regional and central hospital level • Setting forth five key interventions: (i) registration of all births and deaths; (ii) targeted behaviour change of women, their husbands and mothers-in-law; (iii) strengthening health service delivery—focus on SBAs, focus on postnatal care of mother and baby; (iv) service management--mainly about ensuring supplies and logistics; (v) and research focussing on quality of care, and verbal autopsy
National Policy on Skilled Birth Attendant, 2006	
• *Women-friendly services* that are culturally sensitive and affordable to all families, especially those in poor and underserved areas	• Pregnancy and birthing care by an Skilled Birth Attendant [An accredited health professional such as a midwife, doctor or nurse] • Focus on (i) production of SBAs by in-service training and incorporating SBA skills in pre-service curricula of ANM, SN and Doctor training; and (ii) deployment of SBAs to health institutions • Availability of 24 h a day, 7 days a week emergency obstetric care in a close partnership with health workers other than SBAs • Encouraged NGOs and communities to establish community based birthing units • SBA to be supported by: strong referral back-up by a district health team, including supportive supervision; effective partnerships with other health workers, volunteers and TBAs, safety and security
National Safe Motherhood and Newborn Health Long Term Plan (2006–2017)	
• *Equity and women centred care* • *Equity in access and utilisation* of health services for newborn babies including safe motherhood services among the needy • Access embracing financial, institutional and infra-structural factors including, but not limited to, funding, transportation and education; and *positive and welcoming service provider attitudes, trust, honesty, responsiveness, accountability* • *Multi-sectoral approach* as underlying value to address Safe Motherhood and Maternal and Newborn Health (SMNH) issues; the role of other sectors is particularly important in enhancing access and promoting equity • *Women understood not simply as individuals*, but as members of families and communities functioning within complex relationships and social expectations	• Eight strategic outputs to ensure progress in the health of mother and babies: (i) Equity and access: empowerment of individuals, groups and networks with the maternal and newborn care related Behaviour Change Communication (BCC) messages and promotion of birth preparedness and non-discriminatory interpersonal communication between providers and clients; (ii) Delivery of quality maternal and newborn care: 24-h availability of skilled staff with essential drugs and equipment, good community and inter-facility linkages and feedback systems; (iii) Public-private partnership; (iv) Decentralisation: planning and supervising capacity of District Health Office; (v) SBA training; (vi) Information management: collection and use of data according to ethnicity, caste and wealth; and supplement quantitative with qualitative information from; (vii) Physical asset management and procurement; and (viii) Finance such as safety nets for poor and socially excluded
Mother’s Protection Program, Implementation Guideline, 2013	
• Ensure the *right to health as a fundamental constitutional right* of every citizen in accordance with the provision of Nepal’s interim constitution 2006 • *Financial incentives to improve health outcomes*, providing incentives to encourage women to come to institution to have their babies as well as pregnancy check-ups	• The intention of the policy is clear on promoting institutional birth by allocating incentives to women to come to institutions for pregnancy check-ups and birthing; to s*ervice providers* to motivate them to provide birthing care at institutions; and to h*ealth institutions* to encourage them to strengthen birthing and emergency obstetric care • Expands the concept of birthing units by setting specific criteria such as separate birthing room, living apartment for SBA, equipment, 24-h presence of a SBA including a support staff, good referral network, friendly behaviour to woman and her visitors, and the respect of a woman’s privacy • Obstetric reporting to the district and central department of neonatal deaths, stillbirths and babies resuscitated for asphyxia management by each health institution. • Birth or the death registration of a baby, providers receive incentive of home births only if births or deaths are registered by parents
Maternal and Perinatal Death Surveillance and Response (MPDSR), Guideline 2014	
• Value of a life of every mother and every baby; every death can provide information that can result in actions to prevent future maternal and perinatal deaths • Self-reliant and sustainable approach to the improvement of healthcare for women and their babies	• Linking the information system with quality improvement process at a health institution level; real-time monitoring of deaths and assessment of interventions employed. Two main focuses are on: (i) Notification of every death, and (ii) review for further actions to prevent future deaths
Community Based Integrated Management of Neonatal and Childhood Illness (Program Management Module, 2015)	
• Reaching care to disadvantaged and marginalised groups • Provision of quality care through a single integrated package of interventions for newborn and under-five children • Community based care	• Takes into it the lessons from CBNCP, and merges the package with IMCI--thus making a single package for managing newborn and all under-5 years old children’s health problems • Despite the *community based* in its title, still focuses mainly on promotion of institutional births and strengthening of quality of care from health institutions to prevent neonatal deaths • Focus on strengthening the capacity of institutions to manage and treat newborn babies’ complications such as infection, asphyxia and low birth weight • Added a component which describes treatment of baby’s cord infections by using an antiseptic ointment, chlorhexidine • Does not consider management of asphyxia as local health volunteers’ job, which however was considered in previous version—the Community Based Newborn Care • Envisioned developing one to two birthing centres per district to ensure quality referral care for newborns with complications

## Discussion

Nepal’s policies to address perinatal survival since 2000 have been formulated only at the national level. The policies are medically focused, have minimal attention to preventing stillbirths, with a priority on saving newborn babies after birth. The underpinning policy values acknowledge the rhetoric of addressing social determinants of health/health equity and also audit structural determinants such as education, income, ethnicity and geography/ecological differentials in mortality outcomes. All policies have emphasized rural, marginalized and disadvantaged women, but they are not explicit about who these groups are and how they are to be prioritized in actions. Drifting from the core policy values, the strategies focus on accessing and delivering health/medical interventions and health behaviour changes, guided primarily by the intention to promote institutional births. The community based and primary healthcare ideology [39, 40] can therefore be seen to have turned more to being supply-focused, and intended to “correct” communities rather than to engage, sensitize and empower women, families and communities. A brief reflective summary of the policy context is presented in Table 3 that provides take-home messages about what is going on in policy and what could be future considerations (questions) for policy makers as well as researchers.

**Table 3:** A reflective summary of policy context in perinatal survival

Agenda setting	What is going on in policy discourse	Policy considerations (questions) to ask during future policy making
Prevention of stillbirths		
Still not an agenda in policy making, low competing priority	Intention to begin to report stillbirths (occasional statements), but not yet focussed	• Is the technical/epidemiologic separation of stillbirths and newborn death having any social implications? Has this influenced realization of seeing mother and baby as a single unit in any way? Has it affected district/primary healthcare level, how? • Has perinatal survival been considered as an agenda of health promotion, and if so, what could that mean? • Have the policy approach/strategies been community based, empowering individuals and communities, or merely focussed on attempting to correct health behaviours? • Does policy community and implementing units need further realization that perinatal survival is not just a medical issue? • Have health systems (primary health care) been considered to leverage delivery of perinatal healthcare in developing countries? Or are the programmes being implemented just as vertically based technical packages?
Neonatal Survival		
An agenda in policy, healthcare system, but pre-dominantly viewed as a vertical technical/medical initiative	Intention to integrate newborn in child and maternal health within health sector	

Source: Authors’ analysis based on document review

### Policy formulation process: Stakeholders mainly from central level, from only within health sector

Regarding the policy development process, from the present review, first, it is evident that participation in policy formulation was intended primarily within the health sector, within departments such as child and family health, and among government and non-governmental agencies working in the field of maternal and child health. Second, this review notes that the policy documents were developed with consultation among the central level experts. Pradhan and colleagues [41] discuss wider stakeholder involvement in policy making process in formulating Nepal’s National Neonatal Health Strategy. Still, the participatory nature of policy formulation process in the Nepalese policy documents involved are health/medical experts working at government department and non-governmental sector at the central level. Only medical evidence and views of health sector experts are likely to be predominant, as is also evident in the current policies reviewed in this study. It is therefore likely that the experiences of staff working at primary healthcare level and other sectors could be missed. Along with other sectors, the role of implementers (service providers at district and primary healthcare systems) is crucial, as they influence implementation decisions and uptake of care by the clients, also stated as ‘street level bureaucrats’ by Sabatier [42]. It is not just the participation within the health sector which influences population health, but the health system also has stewardship responsibility to work with the wider participation of the sectors beyond it [43]. Only then could policies be helpful in redefining the health systems’ role increasingly towards health promotion and disease prevention, rather than narrowly focused on medicalized services. Otherwise, the focus of the health sector is likely to remain on attending to sick mothers and babies, rather than preventing the occurrence of infections and problems early on.

### Medically focused evidence base: Addressing immediate medical conditions

At the strategy level, Nepal developed a range of policies, plans and guidelines from around the millennium which made as their focus the survival of infants and newborns. Prior to this date, the policies’ key focus was on maternal survival and although some policies mentioned aspects of perinatal survival, the strategic activities predominantly focused on mothers. The change of priority focus to include newborn survival occurred from 2004 onwards with Nepal’s first ever National Neonatal Health Strategy. In describing the intended strategies, the policy documents cite both national and international evidence, with the key focus to prevent immediate medical conditions such as obstetric emergencies, to provide immediate neonatal care and to provide health behaviour change interventions in communities. Nepal’s policies have been up to date in terms of adopting best medical evidence internationally from WHO, UNICEF, USAID and drawing on Nepal’s national demographic and health surveys. The MDG maternal and child survival goals, and the compelling international evidence on introducing SBAs, managing obstetric emergencies, and care and treatment of newborns with infection, complications of low birth weight, and hypothermia have been powerful forces shaping strategic interventions in all of the documents.

### Health service delivery: From community towards health facility focus

Policies outlined strategies in the health system and attempted to address social health behaviours. By setting strategies to mobilize FCHVs for counselling, behaviour change and supportive care during pregnancy, birthing and the postnatal period, policies have prioritized home and community interventions to address lack of preparedness during birth, preventing harmful practices during birthing and newborn care, preventing hypothermia and common infections such as pneumonia and diarrhoea. Policies have prioritized delivery of quality care during pregnancy, delivery and newborn care from health institutions, with quality defined primarily in terms of birthing in institutions, and having birth and postnatal care attended by SBAs. They have also focused significantly on identifying and reviewing the causes of neonatal deaths occurring at institutional level, but not in the community/home- where most of the mountain births take place. Lately, policies have explicitly discouraged home births and incentivized institutional antenatal check-ups and births. Policies have acknowledged the need to address inequitable outcomes in neonatal deaths. Yet, the policy interventions have been informed by medical evidence in reducing neonatal mortality and show little focus on understanding of the sociocultural or geographic contexts of the women and communities on whom they focus, even when documents acknowledge these as important factors. Despite policies outlining home/community, institutions and hospital all as care delivery platforms, the focus remains mainly on changing health related behaviours and attendance at formal institutional care. In this regard, there are inherent contradictions within policy values and strategies. Besides the rhetoric of language in policy values (of “culture, community-based and woman-friendly”), policy strategies remain predominantly within the window of institutionalized and medical care.

The strategies though titled or intended as “community based” do not show any acknowledgement of considering poor perinatal survival in conjunction with Comprehensive Primary Health Care. The authors argue that the health sector has a stewardship role to advocate for consideration of social, cultural and contextual determinants [43] to better address in-country persistent inequities in perinatal survival in Nepal. Only then will the current policy values and the aim of international initiatives such as the Every Newborn Action Plan [44] be realized. The Every Newborn Action Plan aims to end all preventable deaths and has set global targets to reduce stillbirth (per thousand births) and neonatal deaths (per thousand livebirths) to 7 by the end of 2035.

The strategic priority to prevent neonatal mortality at national level has been shaped primarily by the high proportion of homebirths attended by non-skilled attendants. The focus has been on the *system*: improvement in provision of birthing facilities such as birthing units; the *staff*: SBAs as birth attendants; and some aspects of the *consumers*: providing incentives for women to attend formal institutions for pregnancy checks and delivery. The key interventions during pregnancy comprise distribution of iron and anti-worm tablets, Tetanus Toxoid immunisation and introduction of antenatal check-ups. In newborn care, the main focus has been on medical care immediately after birth and postnatally. It is implicit that the policy approach in addressing poor perinatal survival has been viewed mainly as the job of health service providers and health volunteers; the strategies fail to have even an implicit motive to empower women and families and to increase their control or participation over the care and survival pathway. Within the health system itself, besides viewing childbirth and perinatal survival as a medical emergency, to prevent persistent occurrence of perinatal deaths it should be acknowledged first as an agenda of health promotion, as enshrined in the Ottawa Charter for Health Promotion [45]. Genuine actions are needed in policy communities to reorient health services and the role of health service providers, and engage individuals and communities as true partners to prevent deaths which continue to occur due to socio-cultural factors [38, 46, 47].

### Policy target: Only newborn survival, very low focus on preventing stillbirth

Smith and Neupane [48] point out that the issue of newborn survival in Nepal received priority attention due to the country’s commitment to the child health MDGs, leadership, and the policy recognition of newborn health as a problem. However, this priority seemed only to focus on preventing deaths of newborns, while stillbirths have received little focus in the shadow of the competing interest and priority of newborn survival. Within health systems, stillbirths have been considered as a technically separate agenda; stillbirth has also lost social significance as an agenda within the health system itself. Policies simply use the word ‘perinatal’ occasionally, without any intention to focus on the actual issue of perinatal survival. The focus of all policies remains on newborns, with the exception of the MPDSR which does focus on the review of perinatal and maternal deaths. Nevertheless, this is limited mainly to identifying medical causes and avoidable factors to prevent perinatal deaths at institution level. The large number of deaths occurring at home and in communities are not still subjects of such review. The focus has been on preventing death after birth, but paying equal attention to preventing stillbirths would ensure additional care for pregnant women. In addition, it could more strongly help realize the policies’ underpinning values of ‘mother and baby as a single unit’ [49] and ‘continuum of care from pre-pregnancy to up until postnatal period’ [50]. Otherwise, there is the potential to “underplay” stillbirths, making them a low priority in reporting within the health systems, and of low concern in the communities.

### Policy values: Acknowledge rights, socio-cultural contexts

At the value level, one policy – the Maternal and Newborn Health Long Term Plan, 2006 to 2017 [27] - does specifically acknowledge the inequities in neonatal/infant mortalities in terms of geographical location and family income. The National Neonatal Health Strategy identified gender equality and a rights-based approach to ensure newborn survival, while the Mothers’ Protection Programme identifies the right to health and financial safety nets as key policy values to ensure healthcare access. Likewise, MPDSR says it values every mother and every newborn by aiming for a self-reliant and accountable approach of reviewing every perinatal death at hospital level. All policies have intended to address disadvantaged and marginalized populations, but are rarely explicit about who the disadvantaged and specific target groups are. Despite recording obvious differentials in mortality/morbidity rates by region, the policies do not explicitly talk about developing approaches to address these inequitable outcomes on the basis of ecological regions. The exception is providing a slightly higher amount of travel incentive for women from the mountainous regions compared with their hills and plains *(Terai)* counterparts, presumably because they have greater distances to travel to attend health institutions.

A few policies identified woman-friendly care, respect and privacy in health institutions, an equity focus, and focus on socially excluded and under-served regions. The policies have discouraged home births and have shifted their focus significantly to strengthening and delivering quality care from health institutions, and make no mention of how feasible institutional attendance is for the women involved. Although immediate medical/health behaviour change strategies are reasonable at the outset of high prevalence of newborn deaths, this does not empower women, families and communities towards a health promoting approach for care of a mother and baby in a continuum.

The ongoing large numbers of perinatal deaths require immediate priority to identify preventive measures which work. However, since the MDG formulation in 2000, the strategic actions in Nepal have been predominantly medically focussed. If it is to support the survival of every mother and every newborn as aimed for by the WHO in the Every Newborn Action Plan [44], Nepal needs to pay equally serious attention to leveraging these policy values, not just by acknowledging them but by putting actions into workable strategies to address the inequities. The maternal factors, low birth weight and pre-maturity as the causes for large number of perinatal deaths can be prevented only when women and babies are considered as a single unit, when a focus is put on promoting health in a continuum, and when due care is given to the social and cultural contexts in which these deaths continue to occur.

The cultural acceptance and invisibility of neonatal deaths, and largely invisible stillbirths, both within the health system and in communities, also suggest the need for planning strategies to address the related socio-cultural factors. The current government strategies predominantly center on calling women to have institutional births and providing healthcare after birth, but these can prevent babies’ deaths after birth only if women can reach healthcare. These are not sufficient strategies alone to reach out to promote and sensitize women and families at home and in communities where the vulnerabilities to poor health and poor survival originate. The key international calls to improve health, such as the Ottawa Charter for Health Promotion [45], Alma Ata [51] and the WHO Commission on Social Determinants of Health [52], identify strategies which are community led, engaging and empowering women and families, and inter-sectoral-- going beyond the health sector to promote health and to address health inequities. Smith and Neupane [48] discuss the need for priority to be given to newborn survival as a specific agenda within the health sector, which they describe mainly as receiving a technical health/medical priority within a country’s health system. However, we would like to go beyond this, and argue that it is already high time that perinatal death (stillbirth and newborn death) are integrated as a single unit and allocated not just a technical health/medical priority within the health system but also simultaneously consider wider opportunities to sustainably address poor perinatal survival. One approach could be to materialize the underpinning policy values such as ‘women to be understood in complex social context’, ‘women centered care’, ‘socially inclusion’ and ‘rights based care’, as identified in this review. These values should not be treated merely as rhetoric; they should be realized by treating the care and survival of a woman and her baby as a single social whole, not just as a fragmented concept (mother-baby dichotomy) for medical/healthcare. Having this intention within the health system is important because it shapes the behaviours of individuals and communities, and only then can the health system play an effective stewardship role.

### Limitations of this review

One main limitation of this review is that it could not capture how these policy documents have been implemented in practice, and it was also not aimed at capturing the detailed process of policy formulation. Future studies which can illustrate the actual policy process could yield greater insights as to whether the targeted groups, the disadvantaged and marginalized groups, or the key implementing units at district level have been involved, and whether participation in the policy process could become inter-sectoral - as intended within comprehensive primary healthcare - to address social determinants of health/health inequity in relation to perinatal survival. Likewise, a study that can examine how the policy intentions (the values and strategies) have shaped the healthcare system at the district level, as well as the impacts on the women, families and communities in the villages, would provide insights into the effects of these policies on extending healthcare to women, and empowering women and communities, so as to be prepared for more sustainable health promotive and preventive measures in the care and survival of babies.

## Conclusion

This paper has identified the historic timing changes which brought neonatal survival into policy focus in Nepal between 2000 and 2015. Even though the policies have evolved over time to incorporate new evidence and an appropriate shift of focus from maternal survival to maternal *and* newborn survival, they have still not taken account of the many religio-cultural dimensions of maternal survival which are documented in the research literature, or how these factors shape successful pregnancy outcomes and newborn survival. The main content focus has been institutionalized medical care and attempts to correct health behaviour in the communities. The focus of the policies remained on ensuring survival of babies at and after birth; stillbirths still receive very little attention. In terms of a regional focus to address the ongoing high mortality rates, there is no acknowledgement that social context may vary, yet there is very little specific focus on improving neonatal outcomes in the remote mountain areas which record the highest neonatal death rates. Policies also exhibit subtle tensions between institutional versus community focus, and show a mismatch between the underpinning policy values which acknowledge community based, rights-based, inclusion, and social determinants of health/health equity, and yet a strategic orientation focusing predominantly on healthcare/medical interventions confined within health facility premises. This review suggests that future policies in Nepal should integrate stillbirth with neonatal survival, should aim for a separate women and children’s health policy, and develop a policy specific to key ecological/geographic regions. As a further study, it would be imperative to assess whether policy values are not merely stated (acknowledged) in a written document, but rather how these are implemented and impact in practice. Such a study could explain the effect of the policy tension in values and strategic focus in improving perinatal survival in the more disadvantaged regions of the country.

## Abbreviations

ANM: Auxiliary Nurse Midwife
CB-IMCI: Community Based-Integrated Magement of Childhood Illness
CB-IMNCI: Community Based-Integrated Management of Neonatal and Childhood Illness
CBNCP: Community Based Newborn Care
CoIA: Commission on Information and Accountability
EmOC: Emergency Obstetric and Newborn Care
FCHV: Female Community Health Volunteer
FHD: Family Health Division
M/PDR: Maternal/Perinatal Death Review
MCHW: Maternal and Child Health Worker
MPDSR: Maternal and Perintal Death Surveillance and Response
NRs: Nepalese Rupees
NSMLTP: National Safe Motherhood Long Term Plan
NSMNH-LTP: National Safe Motherhood and Newborn Health-Long Term Plan
RHCC: Reproductive Health Coordination Committee
SBA: Skilled Birth Attendant
SDIP: Safe Delivery Incentive Programme

## Footnotes

1 The page refers to the page number of the corresponding document. This also applies to the forthcoming documents in this review.

## References

[1] WHO (2000). Definitions and indicators in family planning maternal & child health and reproductive health used in the WHO regional office for Europe.

[2] WHO. Fact Sheet: Maternal, newborn, child and adolescent Health 2015 [cited 2015 12 July]. Available from: http://www.who.int/maternal_child_adolescent/epidemiology/stillbirth/en/.

[3] WHO. Global Health Observatory (GHO) Data, neonatal mortality: situation and trends 2015 [cited 2016 12 July]. Available from: http://www.who.int/gho/child_health/mortality/neonatal_text/en/.

[4] Lawn JE, Blencowe H, Oza S, You D, Lee AC, Waiswa P (2014). Progress, priorities, and potential beyond survival. The Lancet.

[5] Mason E, McDougall L, Lawn JE, Gupta A, Claeson M, Pillay Y (2014). From evidence to action to deliver a healthy start for the next generation. The Lancet.

[6] NPC (2016). The Millennium Development Goals, Final Status Report, 2000–2015.

[7] UNICEF and WHO. Countdown to 2015 Maternal, newborn and child survival: a decade of tracking Progress for maternal, newborn and child survival, the 2015 report. Geneva, Switzerland: 2015.

[8] MOHP, New ERA, ICF International Inc. Nepal demographic and health survey 2011. Kathmandu, Nepal: Ministry of Health and Population, New ERA, and ICF International, Calverton, Maryland, 2012.

[9] Paudel D, Thapa A, Shedain PR, Paudel B. Trends and Determinants of Neonatal Mortality in Nepal: Further Analysis of the Nepal Demographic and Health Surveys, 2001-2011. Kathmandu, Nepal: 2013 2013. Report No.

[10] MOHP, New ERA, ICF International Inc. Nepal demographic and health survey 2006. Kathmandu, Nepal: Ministry of Health and Population, New ERA, and ICF International, Calverton, Maryland, 2007.

[11] MOHP, New ERA, ICF International Inc. Nepal demographic and health survey 2016. Kathmandu, Nepal: Ministry of Health and Population, New ERA, and ICF International, Calverton, Maryland, 2017.

[12] MOHP, New ERA, ICF International Inc. Nepal demographic and health survey 2016: key indicators report. Kathmandu, Nepal: Ministry of Health and Population, New ERA, and ICF International, Calverton, Maryland, 2017.

[13] UNICEF (2017). Levels and trends in child mortality: report 2017, estimates developed by the UN inter-agency Group for Child Mortality Estimation.

[14] Van Olmen J, Marchal B, Van Damme W, Kegels G, Hill PS (2012). Health systems frameworks in their political context: framing divergent agendas. BMC Public Health.

[15] Walt G, Gilson L (1994). Reforming the health sector in developing countries: the central role of policy analysis. Health Policy Plan.

[16] Hafner T, Shiffman J. The emergence of global attention to health systems strengthening. Health policy and planning. 2012:czs023. 10.1093/heapol/czs023 22407017

[17] Black N, Donald A (2001). Evidence based policy: proceed with careCommentary: research must be taken seriously. Bmj..

[18] Fisher M, Baum FE, Macdougall C, Newman L, Mcdermott D. To what Extent do Australian Health Policy Documents address Social Determinants of Health and Health Equity? Journal of Social Policy. 2016:1–20. 10.1186/s12889-016-3187-6 PMC4908806 27301393

[19] Murray C, Frenk J (2001). World health report 2000: a step towards evidence-based health policy. Lancet.

[20] Paudel M. Socio-cultural and health care contexts of perinatal survival in Rural Mountain villages of Nepal: Flinders University 2017.

[21] Glenton C, Scheel IB, Pradhan S, Lewin S, Hodgins S, Shrestha V (2010). The female community health volunteer programme in Nepal: decision makers’ perceptions of volunteerism, payment and other incentives. Soc Sci Med.

[22] Government of Nepal. National health policy. Kathmandu (Nepal): Ministry of Health and Population, 1991.

[23] Dixit H (2005). Nepal’s quest for health: (health Services of Nepal): educational publishing house.

[24] Ministry of Health and Population [Nepal]. National Reproductive Health Strategy. Kathmandu (Nepal): Deparment of Health Services (Family Health Division), 1998.

[25] Pradhan A, Aryal RH, Regmi G, Ban B, Govindasamy P (1997). Nepal family health survey 1996.

[26] Garenne M, Ronsmans C, Campbell H (1992). The magnitude of mortality from acute respiratory infections in children under 5 years in developing countries. World health statistics quarterly.

[27] MOHP. National Safe Motherhood and Newborn Health Long-Term Plan, 2006–2017, MOHP, Kathmandu (2006). Kathmandu (Nepal): family health division, Department of Health Services, Nepal, 2006.

[28] MOHP. National Neonatal Health Strategy. Kathmandu (Nepal): Department of Health Services (Child Health Division), Ministry of Health and Population (MOHP) Nepal, 2004.

[29] MOHP. National Policy on skilled birth attendants (Supplementatry to safe motherhood policy 1998). Kathmandu (Nepal): Family Health Division, Department of Health Services, Nepal, 2006.

[30] MOHP. Mother’s Protection Program-Implementation Guideline, 2008 (Second Amendment 2013). Kathmandu, Nepal: Department of Health Services (Family Health Division), 2013.

[31] MOHP. Maternal and Perinatal Death Surveillance and Response (MPDSR) Guideline, 2014. Kathmandu, Nepal: Department of Health Services (Family Helath Division), 2014.

[32] DoHS. Community Based Integrated Management of Neonatal and Childhood Illness (Program Management Module). Kathmandu (Nepal): Child Health Division [Department of Health Services], 2015.

[33] MOHP. National Safe Motherhood and Newborn Health Long-Term Plan, 2002–2017, MOHP, Kathmandu (2002). In: family health division DoHS, Nepal, editor. Kathmandu, Nepal: Department of Health Services, Ministry of Health and population (MOHP), Nepal; 2002.

[34] MOHP. Health Sector Strategy: An Agenda for Reform. Kathmandu (Nepal): Ministry of Health (MOH), Nepal; 2004.

[35] Government of Nepal. Nepal Millennium Development Goals Progress Report 2005. In: National Planning Commission N, editor. Kathmandu [Nepal]: National Planning Commission, Nepal; 2005.

[36] MOHP. National Safe Motherhood and Newborn Health Long-Term Plan, 2002–2017, MOHP, Kathmandu (2002). Kathmandu (Nepal): Department of Health Services (family health division), Ministry of Health and population (MOHP), Nepal, 2002.

[37] MOHP. Safe Delivery Incentive Programme, Implementation Guideline 2005 (First amended version 2007). In: Servcies DoH, editor. Kathmandu, Nepal: family health division, Deapartment of Health Services, Nepal; 2007.

[38] Paudel M, Javanparast S, Dasvarma G, Newman L (2018). Religio-cultural factors contributing to perinatal mortality and morbidity in mountain villages of Nepal: Implications for future healthcare provision. PloS one.

[39] Walley J, Lawn JE, Tinker A, De Francisco A, Chopra M, Rudan I (2008). Primary health care: making Alma-Ata a reality. Lancet.

[40] WHO (1978). Primary Health Care: Report of the international conference on primary health care.

[41] Pradhan Y, Upreti SR, K C, N P, Ashish K, Khadka N, Syed U (2012). Newborn survival in Nepal: a decade of change and future implications. Health policy and planning.

[42] Sabatier PA (1991). Toward better theories of the policy process. PS: Political Science & Politics.

[43] CSDH (2008). Closing the gap in a generation: health equity through action on the social determinants of health. Final Report of the Commission on Social Determinants of Health.

[44] WHO. Every newborn: an action plan to end preventable deaths. Geneva, Switzerland: World Health Organization, 2014.

[45] WHO. The Ottawa Charter for Health Promotion: First International Conference on Health Promotion, Ottawa, 21 November 1986 2016 [cited 2016 2 August]. Available from: http://www.who.int/healthpromotion/conferences/previous/ottawa/en/.

[46] Paudel M, Javanparast S, Dasvarma G, Newman L (2018). A qualitative study about the gendered experiences of motherhood and perinatal mortality in mountain villages of Nepal: implications for improving perinatal survival. BMC Pregnancy and Childbirth.

[47] Paudel M, Javanparast S, Newman L, Dasvarma G (2018). Health system barriers influencing perinatal survival in mountain villages of Nepal: implications for future policies and practices. Journal of Health, Population and Nutrition.

[48] Smith SL, Neupane S (2011). Factors in health initiative success: learning from Nepal’s newborn survival initiative. Soc Sci Med.

[49] MOHP. National Safe Motherhood and newborn health long-term plan, 2006–2017. In: family health division DoHS, Nepal, editor. Kathmandu, Nepal: Family Health Division, Department of Health Services, Nepal; 2006.

[50] MOHP. National Neonatal Health Strategy. In: Child Health Division DoHS, Nepal, editor. Kathmandu, Nepal: Department of Health Services (Child Health Division), Ministry of Health and Population (MOHP) Nepal; 2004.

[51] WHO (1978). Declaration of alma ata: Report of the international conference on primary health care.

[52] Solar O, Irwin A (2010). A conceptual framework for action on the social determinants of health. Social Determinants of Health Discussion Paper 2.

[53] Newman L, Baum F, Harris E. ‘Review Framework’, Australian Governments & Health Inequities Project2006 October 07, 2014 [cited 2014 October 07]. Available from: http://som.flinders.edu.au/FUSA/PublicHealth/AHIP/projects_list.htm.

[54] Newman L, Baum F, Harris E (2006). Federal, state and territory government responses to health inequities and the social determinants of health in Australia. Health Promotion Journal of Australia.

